# Revealing factors determining immunodominant responses against dominant epitopes

**DOI:** 10.1007/s00251-019-01134-9

**Published:** 2019-12-06

**Authors:** Wannisa Ritmahan, Can Kesmir, Renske M.A. Vroomans

**Affiliations:** 1 The Centre for Integrative Bioinformatics VU, Vrije Universiteit, Amsterdam, The Netherlands; 2 Theoretical Biology, Utrecht University, Utrecht, The Netherlands; 3 Institute for Advanced Study, University of Amsterdam, Amsterdam, The Netherlands

**Keywords:** Immunodominance (ID), T cell receptor, VDJdb

## Abstract

Upon recognition of peptide-MHC complexes by T cell receptors (TCR), the cognate T cells expand and differentiate into effector T cells to generate protective immunity. Despite the fact that any immune response generates a diverse set of TCR clones against a particular epitope, only a few clones are highly expanded in any immune response. Previous studies observed that the highest frequency clones usually control viral infections better than subdominant clones, but the reasons for this dominance among T cell clones are still unclear. Here, we used publicly available TCR amino acid sequences to study which factors determine whether a response becomes immunodominance (ID) per donor; we classified the largest T cell clone as the epitope-specific dominant clone and all the other clones as subdominant responses (SD). We observed a distinctively hydrophobic CDR3 in ID responses against a dominant epitope from influenza A virus, compared to the SD responses. The common V-J combinations were shared between ID and SD responses, suggesting that the biased V-J recombination events are restricted by epitope specificity; thus, the immunodominance is not directly determined by a bias combination of V and J genetic segments. Our findings reveal a close similarity of global sequence properties between dominant and subdominant clones of epitope-specific responses but detectable distinctive amino acid enrichments in ID. Taken together, we believe this first comparative study of immunodominant and subdominant TCR sequences can guide further studies to resolve factors determining the immunodominance of antiviral as well as tumor-specific T cell responses.

**Electronic supplementary material:**

The online version of this article (10.1007/s00251-019-01134-9) contains supplementary material, which is available to authorized users.

## Introduction

T cells play a crucial role in human adaptive immunity. The recognition of an antigenic peptide bound to the MHC molecule (pMHC) by T cell receptor (TCR) leads to proliferation of the antigen-specific T cell clones (Abbas et al. 2012; Osuna et al. 2014; Pennock et al. 2013). The expanded T cell subsets subsequently differentiate into effector T cells to generate specific immune responses. The effector CD4+ T cells produce several cytokines to activate the recruitment of other immune cells while the effector CD8+ T cells respond to intracellular infections by killing the infected cells. This cascade of events generates long-lived memory T cells that can rapidly initiate the specific immune responses upon re-encountering the same antigen (Abbas et al. 2012).

The TCR is a heterodimer consisting of either a pair of *α* and *β* chains or *γ* and *δ* chains. The *α*/*β* TCR is dominant in human T cell repertoires (Abbas et al. 2012). Each TCR chain is comprised of a variable and a constant extracellular domain. The variable domain is encoded by the germline V, D (only in *β* and *δ* chains) and J genetic segments. Within this domain, the antigen recognition site is formed by three complementarity-determining regions (CDR1, CDR2, and CDR3) (Abbas et al. 2012; Clements et al. 2006; Hou et al. 2016; Hughes et al. 2003). Resolved pMHC-TCR protein structures suggest that mostly CDR3 interacts with pMHC, while the CDR1 and CDR2 are involved in stabilizing the overall TCR-pMHC interaction (Borg et al. 2005; Clements et al. 2006; Dash et al. 2017; Ely et al. 2005; Glanville et al. 2017; Rudolph and Wilson 2002). Thus, the specificity of T cell clones is defined mainly by the CDR3 region of both TCR chains (Danska et al. 1990; Hughes et al. 2003; Tsuchiya et al. 2018; Yassai et al. 2009). The CDR3 region is located at the junctional sites of V(D)-J segments, where recombination of the different genetic segments with the addition and/or removal of nucleotides during imprecise joining generates a highly variable CDR3 sequence (Abbas et al. 2012; Cabaniols et al. 2001; Hou et al. 2016; Pannetier et al. 1993).

A collection of diverse TCR clonotypes generates a unique TCR repertoire in each individual and enables effective protection to a wide range of antigens (Qi et al. 2014; Yassai et al. 2009). Due to a large diversity of T cell repertoires within an individual, several T cells with different TCRs are activated when exposed to the same pMHC complex. Moreover, different individuals having the same infection and same MHC molecules can have a very different responding set of T cell clonotypes (Kedl et al. 2003; Osuna et al. 2014). However, there are usually a few clonotypes that are highly expanded and it is still not fully understood which factors play a role in the selection for large expansion. It is hypothesized that the precursor frequency of naive and memory T cells likely contributes to dominance of T cell clones (Kedl et al. 2003; Kotturi et al. 2008; La Gruta et al. 2006). In addition, the TCR affinity to pMHC (Kedl et al. 2003; Osuna et al. 2014) and antigen dose (La Gruta et al. 2006) have been reported to shape the T cell hierarchy.

In this paper, we make a direct comparison of TCR of immunodominant and subdominant T cell responses to study the role of the composition of TCR in generating immunodominancy. To this end, we analyzed the CDR3 sequences specific to known viral epitopes from the VDJ database (VDJdb) (Shugay et al. 2018). We define an immunodominant clone (ID) as the one having the highest frequency among the CDR3 sequences obtained from one individual. The other CDR3 sequences that are less frequently observed within the same individual are defined as subdominant response (SD). We mainly focused on the CDR3*β* in this study due to data availability and its importance in pMHC recognition. Several common characteristics of CDR3 sequences were analyzed: the CDR3 length, hydropathy, and amino acid arrangement. To our knowledge, this is the first study that makes a direct comparison CDR3 sequences of ID and SD responses.

## Materials and methods

### Data selection from the VDJdb and data processing

The human CDR3 AA sequences were obtained from the VDJdb (https://vdjdb.cdr3.net) (Shugay et al. 2018) on March 2018 in a tab-delimited format. Default search parameters were used, except for origin *Species* (set to “Human”) and selecting both TCR genes (“TRA” and “TRB”). Further filtering of the data was done using an in-house pipeline (available at GitHub repository https://github.com/ritma001/InternshipUU_2017, uses software package R version 3.4.4). First, naive T cell sequences (CD45RA+CCR7+) were removed, as well as sequences with missing frequency because their dominance could not be determined. Because very few CD4+ sequences were present, only CD8+ sequences were used for further analysis. Next, MHC allele groups (Lefranc and Lefranc 2010) and the subgroups of V and J genes (Marsh et al. 2010) were extracted to enable comparison of differently reported values across all studies. Subsequently, CD8+ T cell sequences were divided into subsets based on MHC class and the type of TCR chain (alpha or beta). Several features and physicochemical properties of CDR3 peptides were calculated including whole length, non-VJ length (length of CDR3 region that is not encoded by V and J germline genes), hydropathy, polarity, aliphatic index, bulkiness, net charge, a fraction of acidic AA (“D” and “E”), a fraction of basic AA (“H,” “K,” and “R”), a fraction of aromatic AA (“H,” “F,” “W,” and “Y”), Boman factor (Boman 2003), and 10 Kidera factors (Kidera et al. 1985). The CDR3 encoding performed with custom R scripts and predefined functions from the “alakazam” and “Peptides” R packages.

The paired *α*/*β* data used in this study also derived from the VDJdb by specifying the search parameter *Gene(chain)* as “only paired records.” The downloaded data was processed as previously described and the epitope-specific *α*/*β* pairs were matched using the “complex.id” column.

### Defining ID and SD responses

To extract epitope-specific responses for each individual, we parsed a subject identification and CDR3 clonotype frequency which were provided in the JSON string format. The subject ID was stored in the “meta” column while the frequency can be extracted from the “method” column. Since the subject ID was often missing or insufficient to define an individual response, we combined the original subject.id, replica.id, epitope, reference, and denominator of frequency and used this combination as the subject ID, with “*” as both separator and replacement for missing data. The total clonotype frequency was recalculated if the sum of frequencies in each subject ID was higher than 100%. This was often due to a repetitive measurement of the same CDR3, so this filtering ensured that each clonotype was counted only once in each individual. After correcting for the frequency and sequence redundancy per individual and epitope, the highest CDR3 clone frequency was defined as the ID response while the rest was classified as an SD response. This resulted in a highly imbalanced dataset where for each pMHC combination in an individual, a single ID and several SD were identified. Since it is possible that an ID response of one individual can be an SD response in another individual, it is not trivial to make non-overlapping ID and SD sequence sets. As the number of ID sequences was much smaller than the number of SD sequences (due to our definition of ID and SD), we decided to remove all SD sequences if these sequences were ever found as an ID response in at least one individual in our dataset. In addition, we only selected the CDR3 sequences with a frequency over 0.01% to minimize the bias from very rare clonotypes found in a few publications.

### Naive T cell data

The naive T-cell dataset (3,823 sequences) was derived from two healthy donors from an independent study (Sequence Read Archive, project SRP109035, data is analyzed in collaboration with Peter de Greef). The unique CDR3 clones with an absolute naive count > 1 and not observed among non-naive sequence reads were included. The CDR3 nucleotide sequences were available for these datasets and we implemented a Python (version 3.5.2) script to calculate the non-VJ peptide sequences containing unidentified D segment and random nucleotide insertions.

### Statistical analysis

The frequency distributions were tested for similarity using the Kolmogorov–Smirnov (KS) test. The *P* value was not displayed if the *P* > 0.05 except in the supplementary figures in which “ns” was presented as “non-significant.” The two-sided Mann–Whitney statistics (two-sample Wilcoxon test in R) was used for quantitative comparisons such as length and hydropathy. The same criteria for *P* value was used as previously mentioned. An alternative method other than the two statistics was indicated if performed.

### CDR3 amino acid enrichment analysis

The conserved V and J regions of the CDR3 sequences were removed at the terminal ends. The remaining sequence was defined as “non-VJ” region comprising of encoded D segment and randomly inserted nucleotides. The 20 naturally occurring amino acids were clustered into 3 subgroups based on hydropathic properties including hydrophobic (“A,” “C,” “F,” “I,” “L,” “M,” “W,” and “V”), neutral (“G,” “H,” “P,” “S,” “T,” and “Y”), and hydrophilic (“E,” “D,” “K,” “N,” “Q,” and “R”). These three groups were made following the Kyte–Doolittle hydropathy scale (Kyte and Doolittle 1982).

In order to compare CDR3 sequences varying in length, we mapped the CDR3 sequences to the 2D peptide representative of TCR structure (Lefranc 2014; Lefranc et al. 2003). The CDR3 region is defined as the region delimited by “C” residue at 104 position (C104) and “F”/“W”118 (F/W118) residues. So, the anchored residues (C104 and F/W118) and conserved terminal ends were aligned. According to the IMGT database, 80% of CDR3 are 13-amino acidlong and the added residues are located in the middle of CDR3 with the uniquely defined IMGT positions (Lefranc et al. 2003). To avoid multiple gap insertions in the sequence alignment, we selected CDR3 sequences varying in length from 10 to 15 amino acids which covered > 90% of our dataset. The alignment was performed separately in ID and SD subsets and the entire length of alignment was 17 amino acids due to the maximum CDR3 length (15 amino acids) and the two anchored residues. Next, the positional probability matrix (PPM) was derived for *N* aligned sequences at position *j* where *j* ∈ (1, …, 17):

$$ {PPM}_{k,j}=\frac{1}{N}\sum \limits_{i=1}^N\left({X}_{ij}=k\right) $$

The parameter *i* ∈ (1,…, *N*) indicates the set of sequences, while *k* is a set of 20 amino acids and gap (“-”) and *X*_*ij*_ is the amino acid at position *j*, sequence *i* of the alignment. We then calculated a log ratio of amino acid probability of ID (PMMID) to SD PMM (PMMSD), which we called the “positional log enrichment score” (pLES). A positive ratio indicated the enrichment of amino acids at a certain position in ID compared to SD. Occasional *∞* and − *∞* values appeared in the pLES matrix due to missing amino acids in SD and/or ID. These values were replaced with 5 and – 5, respectively, since the maximum *|pLES|* were observed around 4. The matrix was displayed as a heatmap colored based on pLES. The positive pLES was subsequently converted into a sequence logo for improved visualization.

## Results

**Fig. 1:**
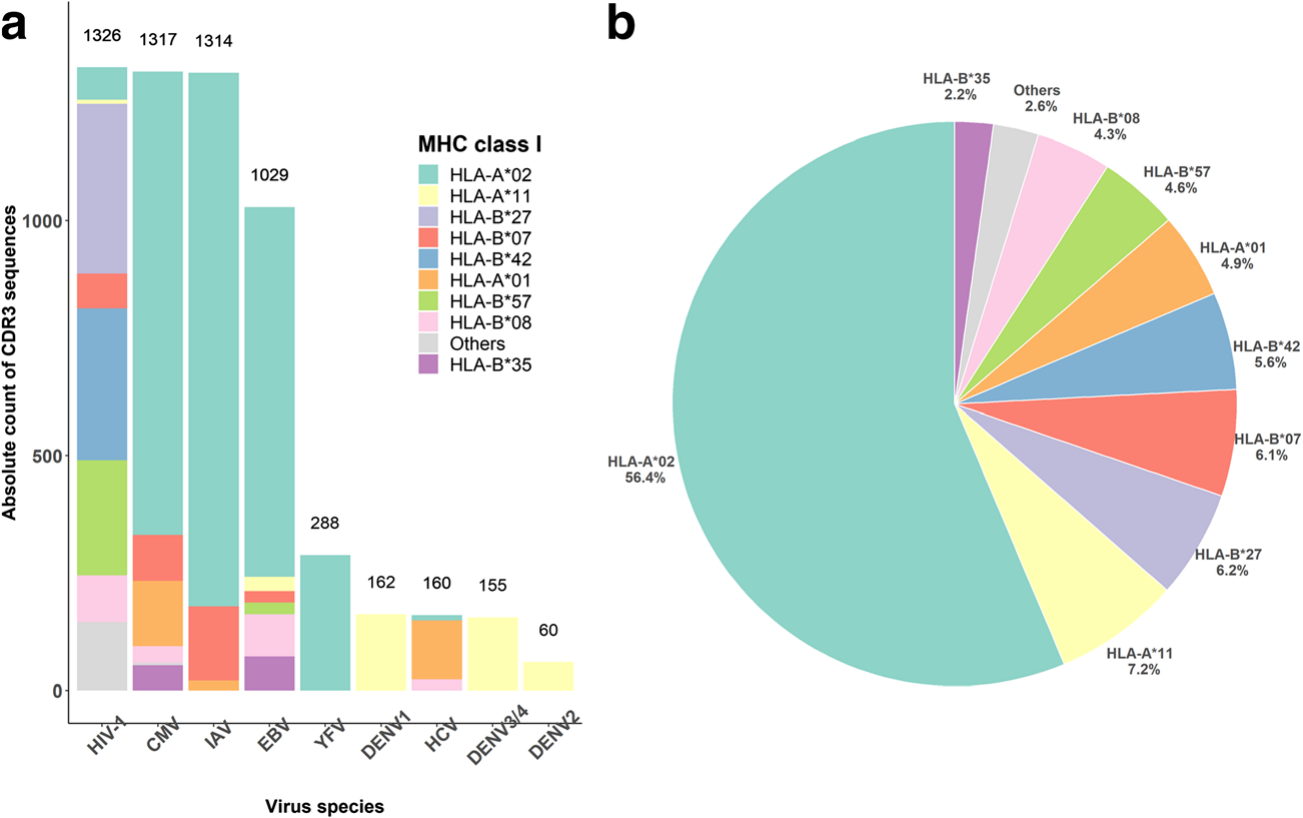
Overview of selected CDR3β sequences from the VDJ db. The human CDR3 sequences (*n* = 5811) against 9 virus species were selected. The CDR3 sequences are color-coded based on the interacting MHC molecule involved in presenting a viral anitgen that is recognised by the CDR3. Different viral antigens derived from the same species can be presented by different MHC molecules (**a**). Most of the selected CDR3 responded to viral presented by HLA-A*02-encoded MHC (**b**)

We obtained human CDR3 sequences from the VDJdb (Shugay et al. 2018), where TCR sequences with known epitope specificity from several studies are collected. We observed 99 (~ 1.7%) identical epitope-specific CDR3*β* detected both as ID and SD responses. This suggests that an ID response from one individual only in very rare cases (< 2%) is an SD response in another individual. In other words, immunodominance of a clone is more universal than originally thought. The redundant CDR3 sequences were removed to obtain a unique CDR3 dataset for ID and SD responses. After this processing, 5811 CDR3 sequences remained which were described as responses against nine virus species, namely HIV-1, CMV, influenza A virus (IAV), EBV, yellow fever virus (YFV), HCV, and four serotypes of dengue virus (DENV1–4) (Fig. 1a). Several epitopes from the same virus were usually presented by different MHC molecules, see, e.g., HIV-1 epitopes (Fig. 1a). As expected, most of the CDR3 sequences (56%, *n* = 3276) were restricted to HLA-A*02 molecules (Fig. 1b). The top three most abundant CDR3 sequences were also A2 restricted: GILGFVFTL (GIL, *n* = 1130) from IAV, NLVPMVATV (NLV, *n* = 979) from CMV, and GLCTLVAML (GLC, *n* = 738) from EBV.

### Influence of CDR3 length on immunodominance

**Fig. 2:**
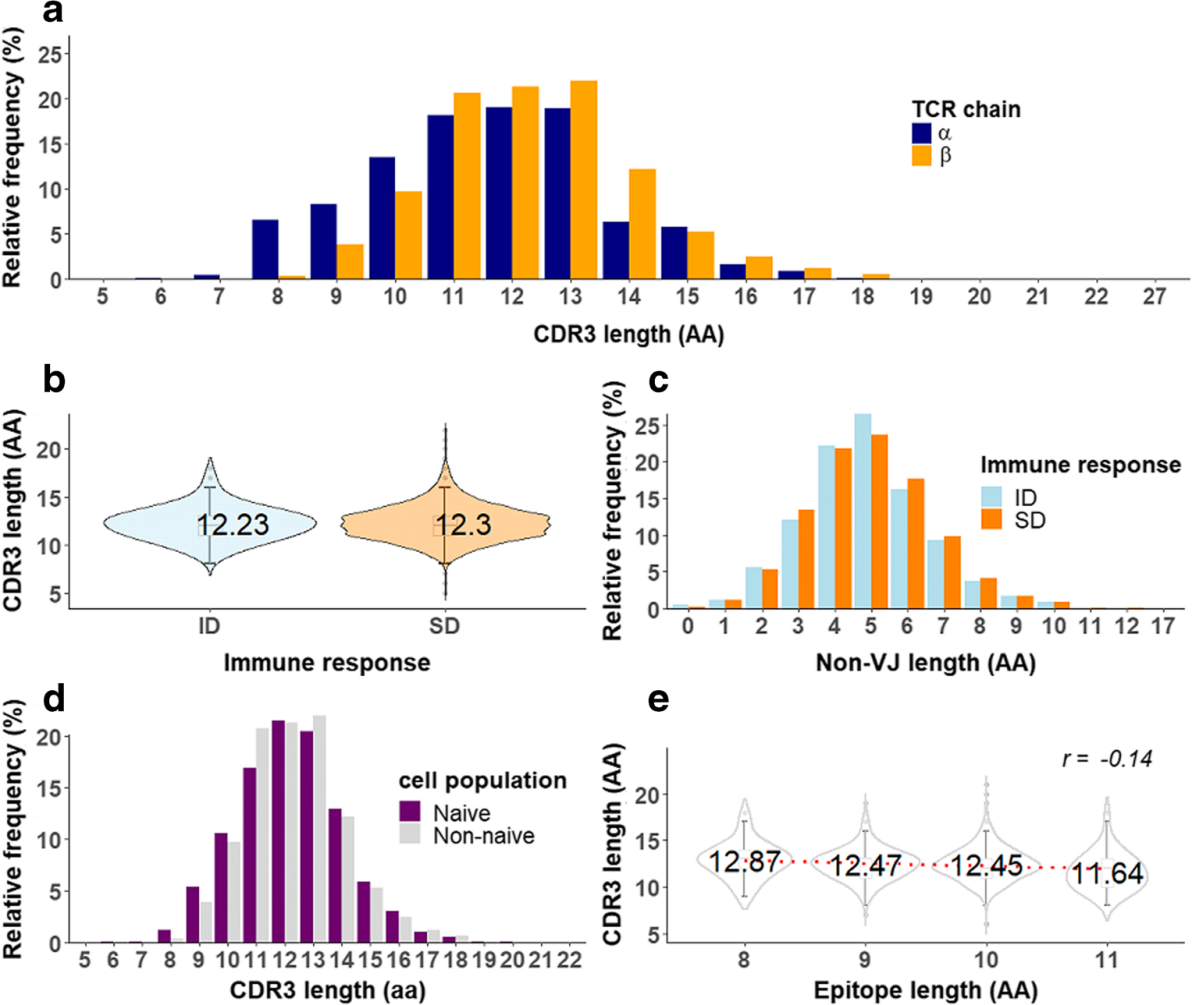
CDR3 length analysis. The distribution of CDR3α (*n* = 2008) and CDR3β (*n* = 5811) lengths are  different (KS test, *P* < 0.0001). The left-shifted CDR3α distribution in relative to the CDR3β suggests a significantly shorter CDR3 in the TCR α chain (**a**). The similar distribution of entire CDR3β length (**b**) and the non-VJ region is observed in ID (*n* = 506) and SD responses (*n* = 5305) (**c**). The CDR3β length comparison between naive T cells (*n* = 3823) and non-naive population reviews significantly longer in the latter (KS test, *P* < 0.005) (**d**). The violin plots of non A2-restricted CDR3β (*n* = 2535) with the mean CDR3β length labels show a negative correlation between the length of epitope and CDR3β (spearman correlation, *r* = − 0.14). The paired comparison between CDR3β length at 8- to 10-amino acid epitopes with the 11-amino acid long confirms the significantly shorter CDR3β in the longer epitope (Mann–Whitney *U* test, all *P* < 0.0001) (**e**)

The CDR3*β* contains the D segment interspersed between V and J regions and therefore should be longer than the CDR3*α*, which lacks the D segment. As expected, the CDR3*β* chains in our dataset were significantly longer than CDR3*α* chains (Fig. 2a, *P* < 0.001 KS test). The length distributions of both chains were normally distributed (Shapiro–Wilk test, *P* < 0.001) as has been observed previously (Moss and Bell 1996; Ma et al. 2016; Niemi et al. 2015). The average lengths of the CDR3*α* and *β* chains were 11.6 *±* 7.4 and 12.3 *±* 5.5 amino acids, respectively. Surprisingly, this result was not always consistent for paired *α*/*β* CDR3, as around 30% of unique *α*/*β* pairs contained longer *α* chains (supplementary Fig. 1A).

Next, we divided each CDR3 sequence into 3 regions; V, J, and non-VJ segments, and performed the length comparison between the *α* and *β* chains. We used the term non-VJ region to refer to the central part of the CDR3 that is not derived from the germline-encoded V and J genes (see the “Materials and methods” section). This junctional region is composed of additional nucleotides and D segment (only in the *β* chain); thus, we expected the longer non-VJ region in the CDR3*β* chain. Interestingly, we observed significantly longer J regions in the CDR3*α* chains while the V and non-VJ segments were longer in CDR3*β* chains (supplementary Fig. 1B–D).

It has been suggested that shorter CDR3 sequences are more likely to be generated, as they closely resemble the encoded peptide and need very deletion events (Hou et al. 2016). To our knowledge, the most concrete factor described so far to explain the response is the precursor frequency (De Boer et al. 2003). Taken together, this suggests that shorter CDR3 sequences are more likely to generate a T cell response. To test this, we compared the length of CDR3β chains between ID (*n* = 506) and SD (*n* = 5305) sequences. As shown in Fig. 2b, CDR3 length was not different between ID and SD responses. Next, we focused on the non-VJ region of CDR3, as this region highly dominates the interaction (Borg et al. 2005; Clements et al. 2006; Dash et al. 2017; Ely et al. 2005; Glanville et al. 2017; Rudolph and Wilson 2002). Almost 75% of the lengths were 3 to 6 amino acids in both ID and SD responses (Fig. 2c) and the ID responses were enriched in shorter regions; however, the difference between the two distributions is not significant, probably due to the small number of ID sequences available.

The ID- and SD-associated CDR3 sequences in our dataset were derived from antigen-experienced T cells (non-naive T cells), and therefore, it might be expected that they share a similar distribution of CDR3 lengths. To examine the difference between non-naive and naive T cells in their CDR3 length distribution, we compared the sequences in our dataset (ID and SD combined) with CDR3 lengths of the naive CD8+ T cell β chain (see the “Materials and methods” section). The average length of naive CDR3 was the same as non-naive CDR3s (12.3 amino acids); however, the distribution of the CDR3 length was significantly different between the two populations (Fig. 2d): longer CDR3 were somewhat enriched in the non-naive population.

Even after grouping CDR3 sequences based on the MHC molecules to which they are restricted, or the epitopes they recognize, we did not observe a difference between the CDR3 length of ID and SD (supplementary Fig. 2A, B). Interestingly, we did observe a negative relationship between the length of epitope and the corresponding CDR3 sequences. This result might reflect a bias due to the dominance of A2 restricted T cell responses in our dataset (Fig. 1b) and the A2 epitopes were all 9 amino acid long. We removed A2 epitopes and we still found a weak but significant negative correlation (Spearman correlation coefficient, *r* = − 0.14, *P* < 0.001) between epitope length and CDR3 length (Fig. 2e). This trend was also observed in a set with only EBV and HIV-1 epitopes (8 to 11 amino acid long), restricted by different HLA types (supplementary Fig. 2C). This result suggests that longer CDR3β chains are not needed to recognize longer epitopes bulging from the MHC molecule, which was previously hypothesized/shown (Ekeruche-Makinde et al. 2013). Next, we compared epitope length with the combined CDR3 lengths of paired *α*/*β* CDR3 chains (no A2 epitopes included; supplementary Fig. 2D). The significant correlation we found between the epitope length and CDR3 length disappeared in this case, which might suggest that a shorter CDR3β chain may be compensated with a longer CDR3α chain to preserve the interaction with varying epitope lengths (supplementary Fig. 2E).

### Amino acid composition in CDR3 sequences

**Fig. 3:**
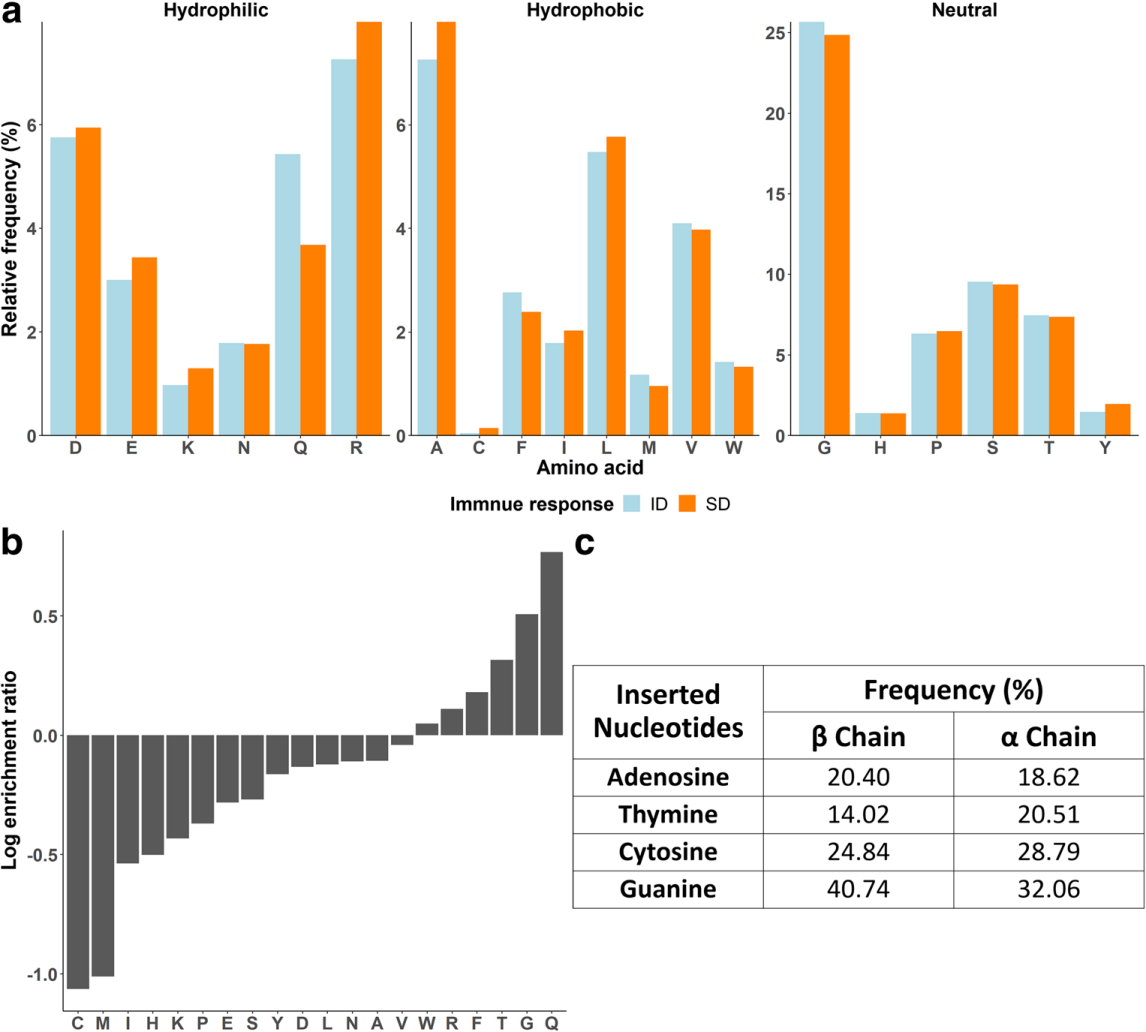
Amino acid composition of the non-VJ region. The hydrophilic, hydrophobic, and neutral residues distributions differ between ID and SD responses (KS test, *P* < 0.002, *P* < 0.003, and *P* < 0.03, respectively). The neutral amino acids are predominant in the non-VJ region and the “G” is the most enriched residue (**a**). The log ratio of amino acid frequency in the non-VJ CDR3β to CDR3α demonstrates highly enriched “Q” in CDR3β and hydrophobic amino acids, namely, “C,” “M,” and “I” in CDR3α (**b**). Guanine is frequently observed in non-VJ of naive CDR3α and CDR3β nucleotide sequences (**c**)

The N- and C-terminus of CDR3 sequences are highly conserved due to the germline encoded V and J segments, respectively. However, the centrally variable region could hold the potential to diverse immune responses. Therefore, we selectively studied amino acid profiles of the non-VJ region of CDR3β sequences. The ID and SD responses differed slightly in their different amino acid distribution (Fig. 3a). In general, the frequently presented amino acids were small (based on the molecular volume) and neutral: glycine (“G”), serine (“S”), threonine (“T”), and proline (“P”). Additionally, arginine (“R”), glutamine (“Q”), glutamic acid (“D”), alanine (“A”), and leucine (“L”) were observed more than the expected 5%. Approximately 25% of amino acids detected in the non V-J region is “G” irrespective of being an ID or SD response. This observation was consistent across the HLA alleles (supplementary Fig. 3A). The highly enriched “G” residue in CDR3β sequences is possibly due to the guanine-rich nucleotide sequences of D segment which was estimated to be around 70% in both D1 and D2 genes (Freeman et al. 2009; Venturi et al. 2008). Therefore, the D segment might cause a bias in codons containing guanine (“g”) like “ggx” (where x stands for any nucleotide) for “G.” If this is the case, the dominance of the glycine residue should disappear in CDR3α sequences that contain no D segment. To test this hypothesis, we compared the amino acid distributions of all CDR3 α and β sequences in our dataset (Fig. 3b). Some hydrophobic residues, namely, cysteine (“C”), methionine (“M”), and isoleucine (“I”) are highly enriched in the non-VJ region of CDR3α relative to the β chain (Fig. 3b). However, the most predominant residue of the CDR3α was also “G.” The log enrichment ratio between the CDR3β and CDR3α chains of “G” was positive suggesting that the residue in CDR3β was more frequently observed than the CDR3α. However, the difference is rather small making it unlikely that the D segment is solely responsible for the enrichment of “G” in CDR3β sequences (Fig. 3b).

To determine if the non-VJ segment of the antigen experienced T cells are shaped by naive T cells, we also compared the amino acid composition of the naive and antigen experienced CDR3 population. The amino acid profiles of CDR3β and CDR3α sequences were similar in both T cell populations (supplementary Fig. 3B). In general, the five most frequent amino acids (S, R, P, L, and G) observed in both CDR3 chains are the ones that can be encoded by 4 or more codons, suggesting an effect of codon degeneracy on frequency of an amino acid in non-VJ regions. The nucleotide sequences available for the naive T cell data confirmed enriched guanine even without the D segment in CDR3α (Fig. 3c). Therefore, we can conclude that enrichment of the nucleotide guanine (x), which probably results in an enrichment of the amino acid glycine (due to ggx code, see above) is not influenced by D segment, TCR chain, or antigen exposure.

To test position specific enrichment of amino acids in CDR3 sequences, we aligned unique CDR3 sequences of ID and SD separately. Prior to the alignment, CDR3 sequences were mapped to the IMGT positions corresponding to the 2D peptide chains representing the functional TCR structure (Lefranc 2014; Lefranc et al. 2003). As a consequence, the conserved terminal ends were aligned and gaps were allowed in the central variable domain. We then created the positional probabilistic matrices (PPM) to present the observed frequency of each amino acid throughout the CDR3 alignment. From this PPM, we also calculated positional probability ratios of ID to SD responses. This log ratio value was used as a positional log enrichment score (see the “Materials and methods” section). In most of the positions, all amino acids were presented equally in ID and SD. However, in positions 106, 112.1, and 115 to 117, several amino acids were enriched in SD sequences, suggesting that certain amino acids promote the subdominance probably because of the weak interactions with pMHC complexes (supplementary Fig. 4).

**Fig. 4:**
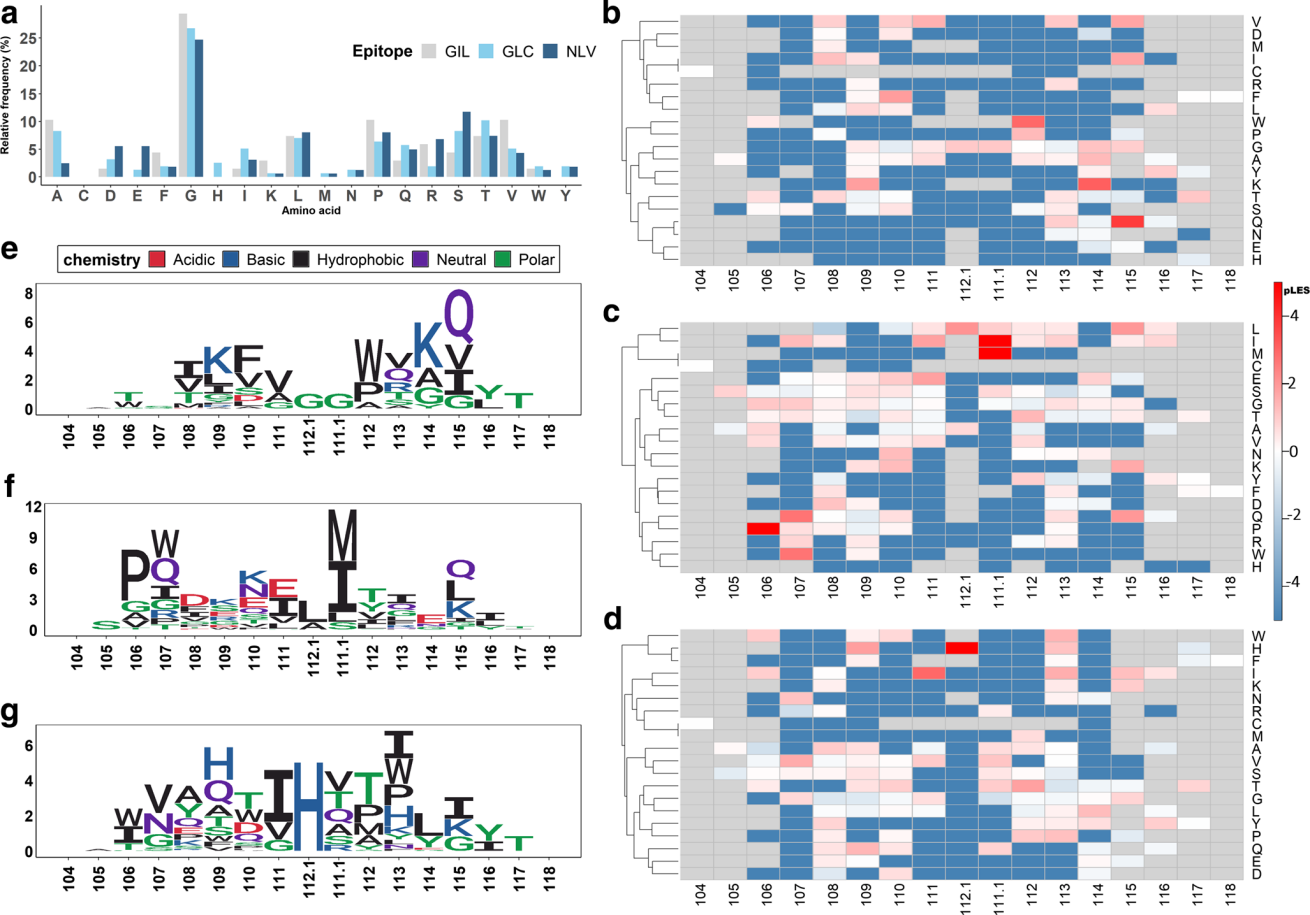
pLES heatmaps and corresponding sequence logos of CDR3 against the three prevalent epitopes: GIL, GLC, and NLV. Amino acid distributions (ID and SD combined) of CDR3 sequences against the three epitopes are similar (tested by Anderson–Darling *k*-sample test (Canada 1986)) (**a**). Log ratio of amino acid frequency in ID to SD at each CDR3 position from C104 to W/F118 are displayed in pLES heatmap. The positive (red) indicates enriched ID and the negative (blue) referred to enrichment in SD relative to ID. The gray color illustrates the absence of an amino acid at a certain position. GIL, GLC, and NLV (**b**–**d**) were converted into sequence logos which were created from positive pLES from antigen-specific CDR3 alignment (**e**–**g**)

To refine the possible motifs in analysis, we repeated the same procedure at the level (Fig. 4a). We selected three with the most CDR3 sequences (GIL, NLV, and GLC) in order to have enough data. All three were presented by the HLA-A*02 molecule. The SD-associated CDR3 sequences were abundant and only a small fraction of around 2–6% of CDR3 sequences were defined as ID for each epitope. We computed the matrices for each and displayed the matrices as heat maps and sequence logos (Fig. 4b–g). The negative (blue), inferring an enrichment in SD, was observed dominantly (Fig. 4b–d), while few highly enriched amino acids were found in ID (red) responses. The sequence logos of the positive amino acids (Fig. 4e–g) displayed very different patterns for the different, suggesting that a simple motif/pattern is very difficult to find even among CDR3 sequences recognizing different on the same HLA molecule. Although one can detect some distinctive patterns between ID and SD per epitope, a general amino acid signature that defines CDR3 being an ID response was not found.

## Discussion

The recently developed VDJdb contains a vast amount of epitope-specific CDR3 sequences. These sequences were derived from multiple research groups working on shared CDR3 sequences between individuals, recognition of dominant epitopes, binding motifs in CDR3 region, V(D)-J recombinations, and repertoire diversity (Shugay et al. 2018). To our knowledge, the immunodominancy of T cell responses had not yet been explicitly addressed with these data, despite its importance in shaping immune responses (Osuna et al. 2014). We here present the first attempt to do this.

We first focused on common properties like the length and of CDR3β sequences, since they have been shown to impact immune responses (Hou et al. 2016; Ma et al. 2016; Niemi et al. 2015; Stadinski et al. 2016; Wang et al. 2017). As expected, these two properties are insufficient for explaining why some responses are while others are subdominant. However, we found other interesting correlations. For instance, longer and more hydrophobic CDR3 are enriched among non-naive T cells, and there is a negative correlation between the epitope length and CDR3 length. Furthermore, CDR3 restricted by the HLA-A locus were more hydrophobic than those restricted to the B locus. We believed that these global descriptions of CDR3 sequence properties, despite being unrelated to this study, shed light on future immunology research in several aspects.

Focusing on the CDR3 responses only for HLA-A2 restricted GIL, we found significantly higher CDR3 in ID responses compared to SD responses. We observedthe CDR3 clones responding to GIL containing more hydrophobic residues than that of subdominant clones (data not shown). Previous studies on the GIL-MHC complex discovered a plain interacting site with fewer protruding side chains of the CDR3 sequence (Chen et al. 2017). This structural constraint could limit the selection of CDR3 sequences to those that are more hydrophobic, explaining our finding of higher CDR3 in ID responses against GIL. For all the other responses, we did not find such a clear effect. 

We consistently observed high enrichment of glycine in the non-VJ region regardless of antigen exposure and CDR3 chains. Apart from the highly predominant glycine, different amino acid profiles were observed in epitope-specific CDR3, making it impossible to identify a common amino acid motif/pattern indicator of immunodominancy in CDR data. This result currently suggests that immunodominancy is mostly determined by specific TCR-epitope interaction properties, although with more data a common immunodominance motif might still be identified.

Another possible contributing factor shaping immunodominance is the bias in V-J recombinations which has been observed in many studies (Chen et al. 2017; Freeman et al. 2009; Moss and Bell 1996; Lundegaard et al. 2010; Ma et al. 2016; Song et al. 2017). We observed uniquely biased V-J usage for each epitope and found that the combined V and J genes in ID clones were largely shared with the SD responses (data not shown). To predict the generation probabilities of ID and SD responses (based on their VDJ recombinations), we made use of the OLGA server (Sethna et al. 2019). A per-epitope comparison of the generation probabilities for ID and SD responses did not indicate a significant difference, suggesting that the immunodominance of a T cell clone cannot be explained by high generation probability of its TCR sequence.

We mainly performed our analysis on CDR3β and when available on CDR3α sequences. However, the binding site of TCR to pMHC is composed of CDR1, CDR 2, and CDR3 regions from both TCR chains (Abbas et al. 2012; Clements et al. 2006; Hou et al. 2016; Hughes et al. 2003). The entire complex interaction can therefore not be studied completely using the available data on CDR3 sequences, if the other CDR regions play unexpectedly pivotal role in TCR-pMHC engagement (Miyazawa and Jernigan 1996). Thus, our analysis provides only a preliminary view of the interaction between CDR3 and bounded epitopes. When more data become available on CDR2 and CDR3 sequences, our analysis should be repeated.

In conclusion, we found that the global properties of CDR3 sequences between ID and SD are highly similar. However, several features are distinctive regarding epitope specificity and, thus, can enable classification of epitope-specific CDR3 from a diverse T cell response. Most interesting findings of our study, though slightly outside of our initial research question, were differences between antigen experienced and naive T cell clones. The results raised by our analysis ask for larger sets of naive T cell repertoires for validation.

## Electronic supplementary material

ESM 1 (DOCX 410 kb)
