# Supplementary material for: Revealing factors determining immunodominant responses against dominant epitopes
Source: Immunogenetics. 2019 Dec 6;72(1):109–18. doi: 10.1007/s00251-019-01134-9 (PMC6971151; doi:10.1007/s00251-019-01134-9)
Supplement: Supplementary file 1 — (DOCX 410 kb) [file 251_2019_1134_MOESM1_ESM.docx]

**Supplementary Figures**


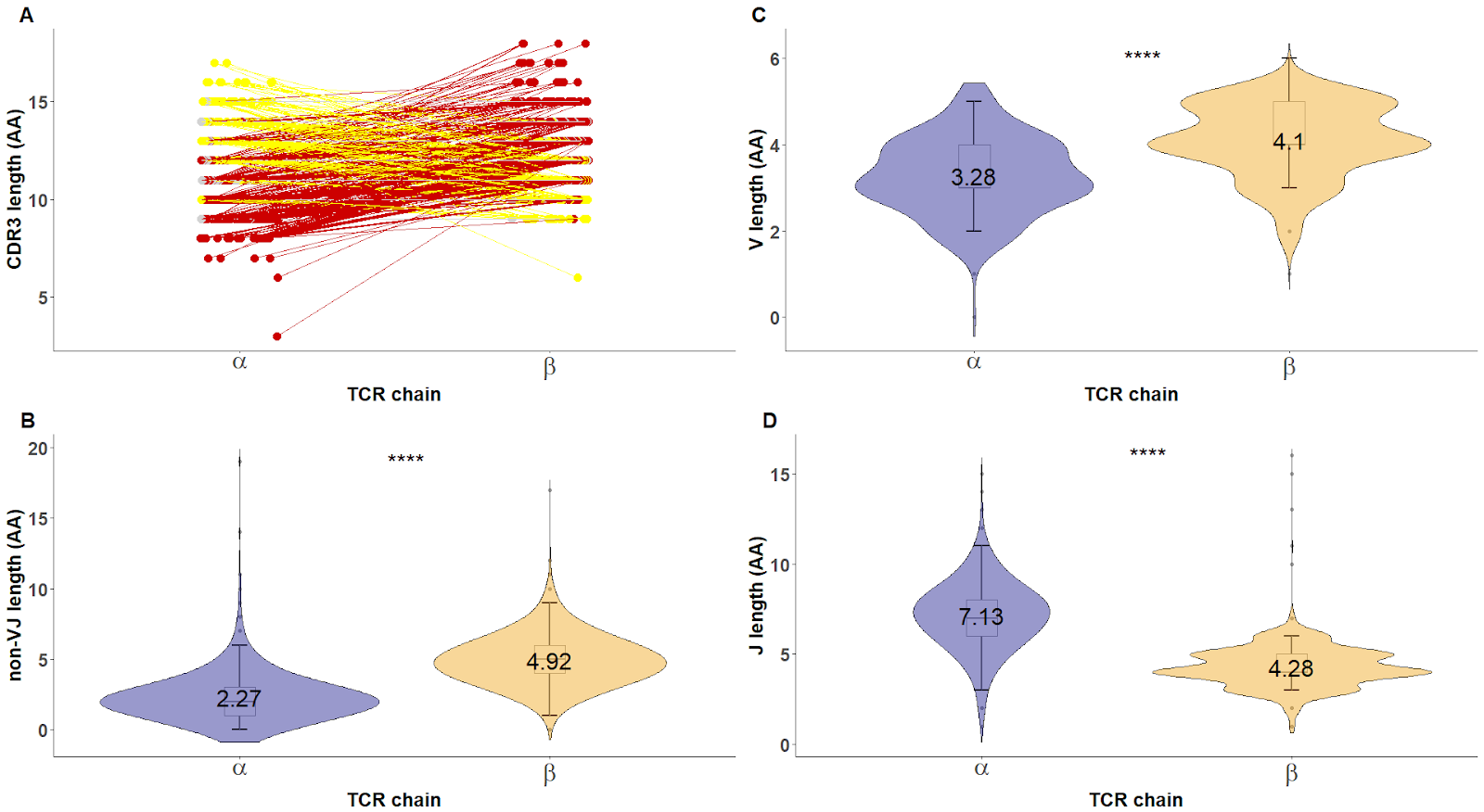


**Supplementary Figure 1| Extension of CDR3 length analysis on paired α/β CDR3.** CDR3 length comparisons between unique pair of α/β CDR3 in which the **red**, **yellow** and **gray** colors represented a pair with **longer CDR3β**, **longer CDR3α** and **equal lengths**, respectively (**A**).  The V and non-VJ region were significant longer in CDR3β (**C**, **B**) while the J region of CDR3β was shorter than CDR3α (**D**).


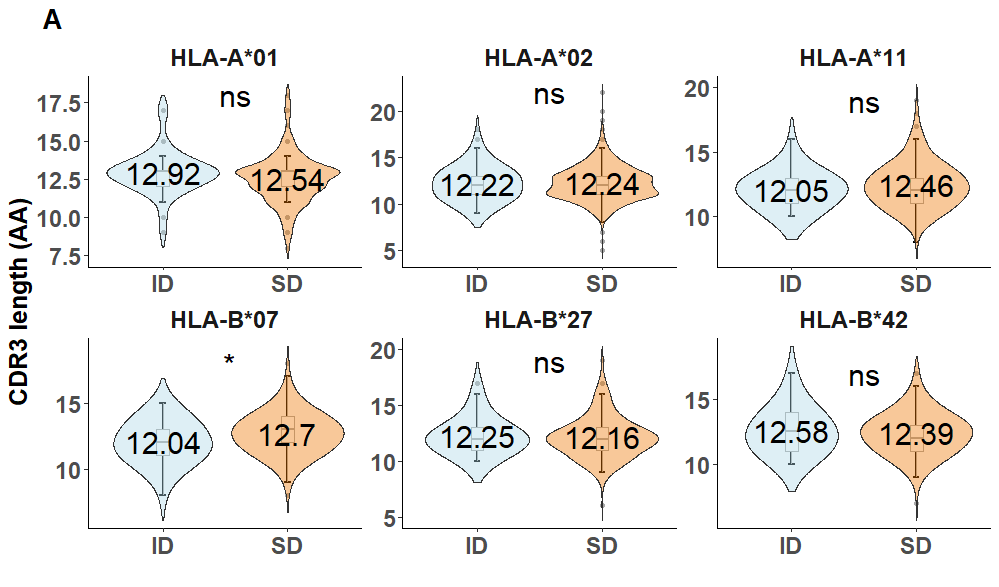


**Supplementary Figure 2A| Extension of CDR3 length analysis per (only for 6 MHC molecules with most TCR responses, *n=5811*).** The significantly shorter CDR3 length in ID was only observed in HLA-B*07 dataset (*n=354*). This dataset contained 6 epitopes from Influenza-A virus (IAV), HIV-1, CMV and EBV.


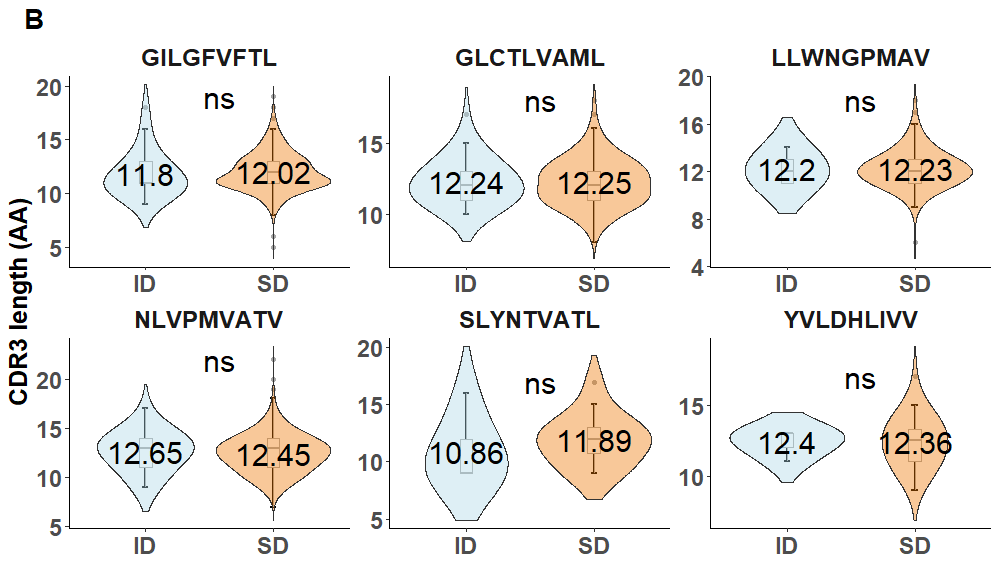


**Supplementary Figure 2B| Extension of CDR3 length analysis on the six viral epitopes from HLA-A2 restricted responses (*n=3276*).** The six epitopes were GILGFVFTL from IAV (*ID=25, SD=1105*),  NLVPMVATV from CMV (*ID=37, SD=942*),  GLCTLVAML from EBV (*ID=42, SD=696*), LLWNGPMAV from YFV (*ID=5, SD=283*),  YVLDHLIVV from EBV (*ID=5, SD=44*) and SLYNTVATL from HIV-1 from (*ID=7, SD=28*).


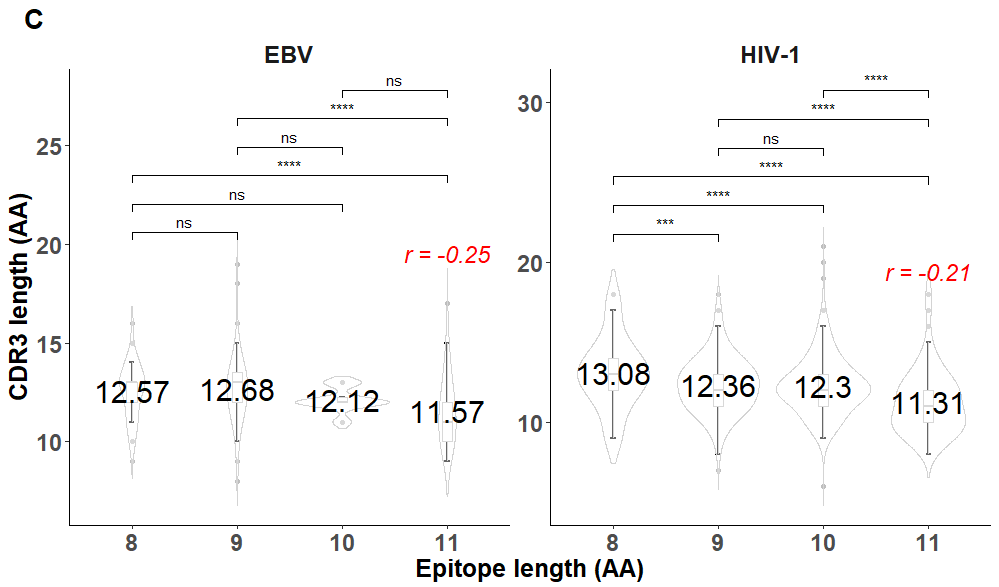


**Supplementary Figure 2C| Relationship between the length of CDR3 and their corresponding epitopes from EBV and HIV-1 across multiple MHCs.** For this analysis we removed all HLA-A2 restricted TCR sequences.


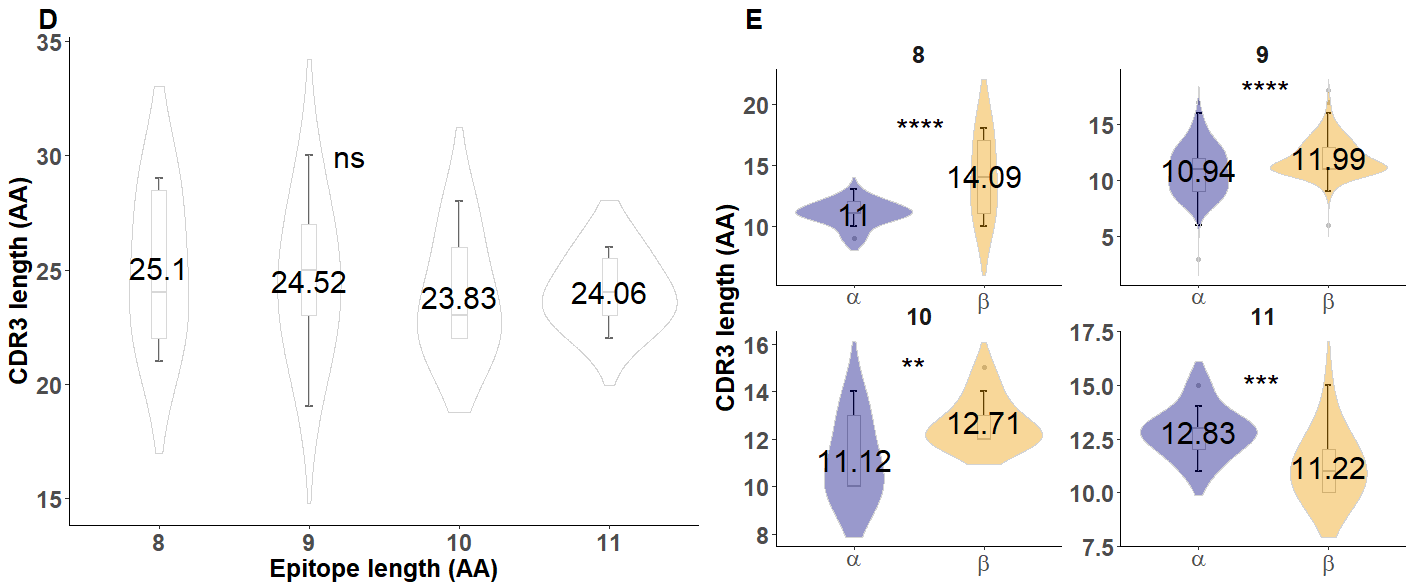


**Supplementary Figure 2D-E| Extension of CDR3 length analysis on paired  α/βCDR3 excluding A2 restricted responses (*n=96*).** The sum of paired CDR3 length (AA) was similar across different epitope length (*Kruskal-Wallis test, P > 0.05*) (**D**). However, the significantly different CDR3 lengths between paired α/βCDR3 per epitope length were seen (*Wilcoxon test, P<0.01* in all cases) (**E**).


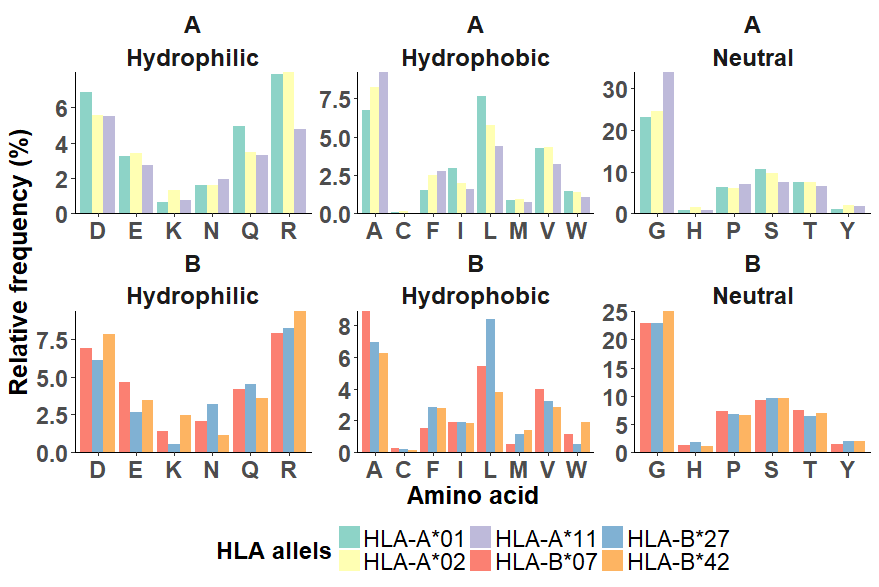


**Supplementary Figure 3A| AA composition of the non-VJ region across the top six MHC of CDR3β.** The top and bottom rows represented MHC encoded from A and B loci, respectively. No statistical test was performed but, consistently enriched in neutral AA and predominant “G” were observed.


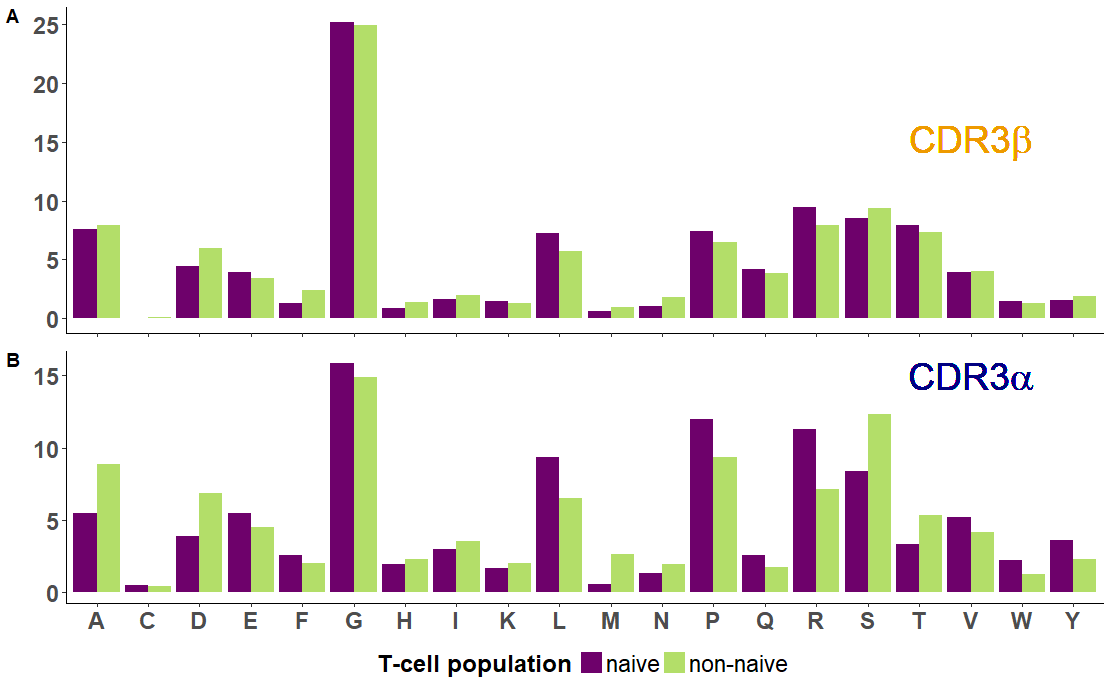


**Supplementary Figure 3B| Similarity between AA composition of the non-VJ region between naive and non-naive (ID and SD combined) CDR3α and CDR3β.** “G” was predominantly observed regarding the TCR chains and T-cell population but, it is more enriched in the CDR3β due to extra D segment.


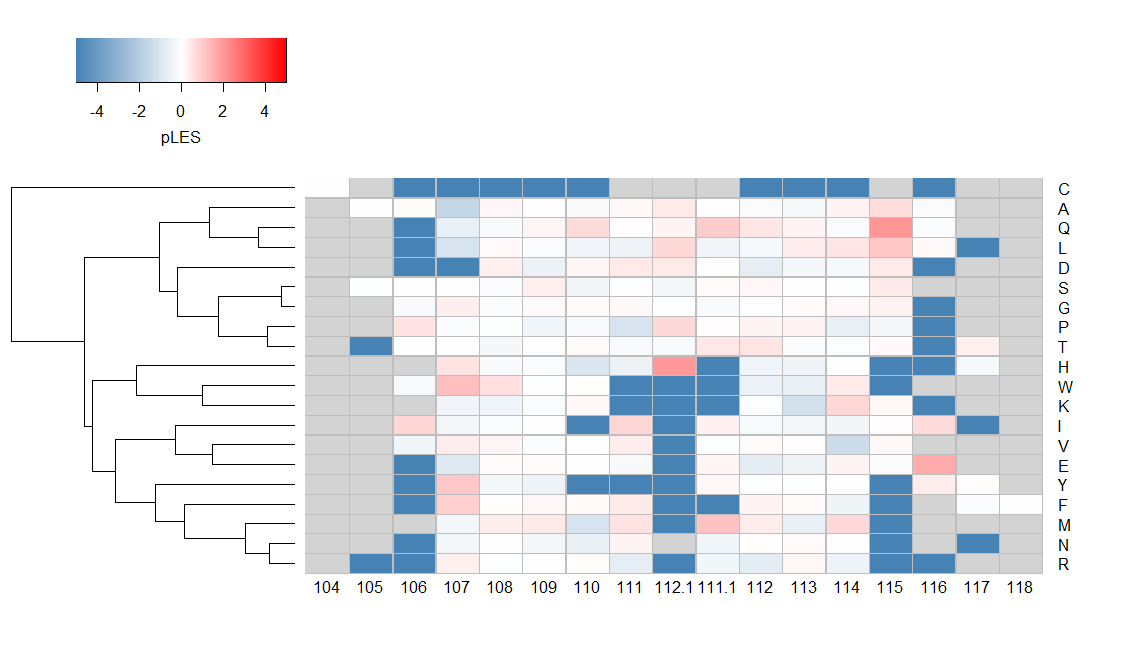


**Suplementary Figure 4 Heatmap of pLES matrix calculated from entire CDR3β containing several epitopes.** Most of AA were equally presented in ID and SD responses (white color, pLES = 0) while enrichment of specific AA in SD as indicated by the negative pLES (blue) were observed at 106, 112.1, 115 to 117. “Q” at 107, “E” at 117 and “H” at 112.1 were weakly enriched in ID (red) in relative to SD.
